# The dual role of ATF4 in neurons: from stress adaptation to therapeutic intervention

**DOI:** 10.3389/fnmol.2026.1816333

**Published:** 2026-06-11

**Authors:** Hua Huang, Yao Xu, Xingyi Li, Chenggang Li, Wuxue Peng

**Affiliations:** Fengdu General Hospital, Chongqing, China

**Keywords:** ATF4, homeostatic rheostat, integrated stress response, neurodegeneration, synaptic plasticity

## Abstract

Activating transcription factor 4 (ATF4) functions as the central transcriptional arbiter of the integrated stress response (ISR) in neurons. Its translation is gated by diverse upstream kinases via the eIF2α pathway, while its functional output is critically shaped by context-dependent interactions with specific protein partners (e.g., C/EBPβ or CHOP). We conceptualize ATF4 as a spatiotemporal rheostat whose regulatory mandate is stage-specific: it acts as a physiological switch during neocortical development and maintains synaptic and mitochondrial integrity in the adult brain. However, this precise regulation fails in neurological disorders, including Alzheimer’s disease, Parkinson’s disease, cerebral ischemia, and epilepsy. Chronic, maladaptive ATF4 signaling—often driven by pathological heterodimerization—catalyzes neuroinflammation, ferroptosis, and circuit failure. Crucially, contemporary challenges in clinical translation highlight a “therapeutic paradox,” where broad or untimely pathway inhibition may inadvertently dismantle essential neuroprotective shields. We therefore advocate for a paradigm shift toward “kinetic recalibration”—the development of precision interventions designed to restore the proteostatic and information-processing homeostasis of the stressed nervous system.

## 1 Introduction

Activating transcription factor 4 (ATF4) is a master orchestrator of the integrated stress response (ISR), tasked with translating diverse cellular insults—such as endoplasmic reticulum (ER) stress and mitochondrial dysfunction—into specific transcriptional outputs in neurons (Pakos-Zebrucka et al., 2016). Characterized by minimal basal expression and rapid stress-induced kinetics, ATF4 acts as a critical sensor for the proteostatic imbalances that hallmark neurodegenerative pathology (Gachon et al., 2001; Tang H. et al., 2024). Neurons, given their post-mitotic nature and high metabolic demand, are uniquely susceptible to such proteotoxic stress. This response is primarily orchestrated by the protein kinase RNA-like endoplasmic reticulum kinase (PERK), which phosphorylates eukaryotic initiation factor 2 alpha (eIF2α) to selectively bypass global translational inhibition and promote ATF4 synthesis. Consequently, the PERK-eIF2α-ATF4 axis occupies a central position in determining the neuronal survival-death equilibrium across various pathological contexts (Harding et al., 2003; Pitale et al., 2017; Rozpedek et al., 2015; Wei et al., 2021; Xu et al., 2023; Yang et al., 2019, 2023).

While traditionally viewed through the lens of acute stress adaptation, ATF4 is increasingly recognized as a spatiotemporal regulator of basal neuronal physiology. It integrates metabolic flux by regulating enzymes such as phosphoenolpyruvate carboxykinase 2 (PCK2), thereby rerouting neuronal energy supplies to maintain energetic homeostasis under fluctuating nutrient demands (Chen et al., 2025). In the mature brain, ATF4 constrains synaptic potency and memory formation by gating neuronal bioenergetic capacity (Mahmood et al., 2024). Its stoichiometry is also tightly linked to psychiatric health; for instance, nuclear overabundance of ATF4, driven by mutations in Disrupted-in-Schizophrenia 1 (DISC1) mutations, provides a mechanistic link to synaptic failure in schizophrenia (Wang et al., 2021). The “dual-edged” nature of ATF4 is starkly evident in conditions like Parkinson’s disease, where it can facilitate either Parkin-mediated neuroprotection or neurotoxin-induced apoptosis depending on the specific stress context (Aimé et al., 2020; Demmings et al., 2021; Sun et al., 2013).

Despite extensive research, the molecular logic governing how ATF4 discriminates between adaptive and maladaptive outputs remains elusive. This review moves beyond a general summary of the ISR to specifically delineate ATF4 as a molecular rheostat in adult neurons. We focus on elucidating how transcriptional interactors and contextual metabolic signals steer ATF4-dependent fate decisions. Furthermore, we provide a critical synthesis of recent clinical efforts—and setbacks, such as those involving eukaryotic initiation factor 2B (eIF2B) modulators—in targeting this axis. By defining these distinct molecular thresholds, we aim to clarify why wholesale ISR modulation has faced translational challenges and to propose precision, context-aware strategies for next-generation neurotherapeutics.

## 2 Expression and regulatory mechanisms of ATF4 in neurons

### 2.1 The PERK-eIF2α axis: a central gatekeeper of ATF4 induction

**FIGURE 1:**
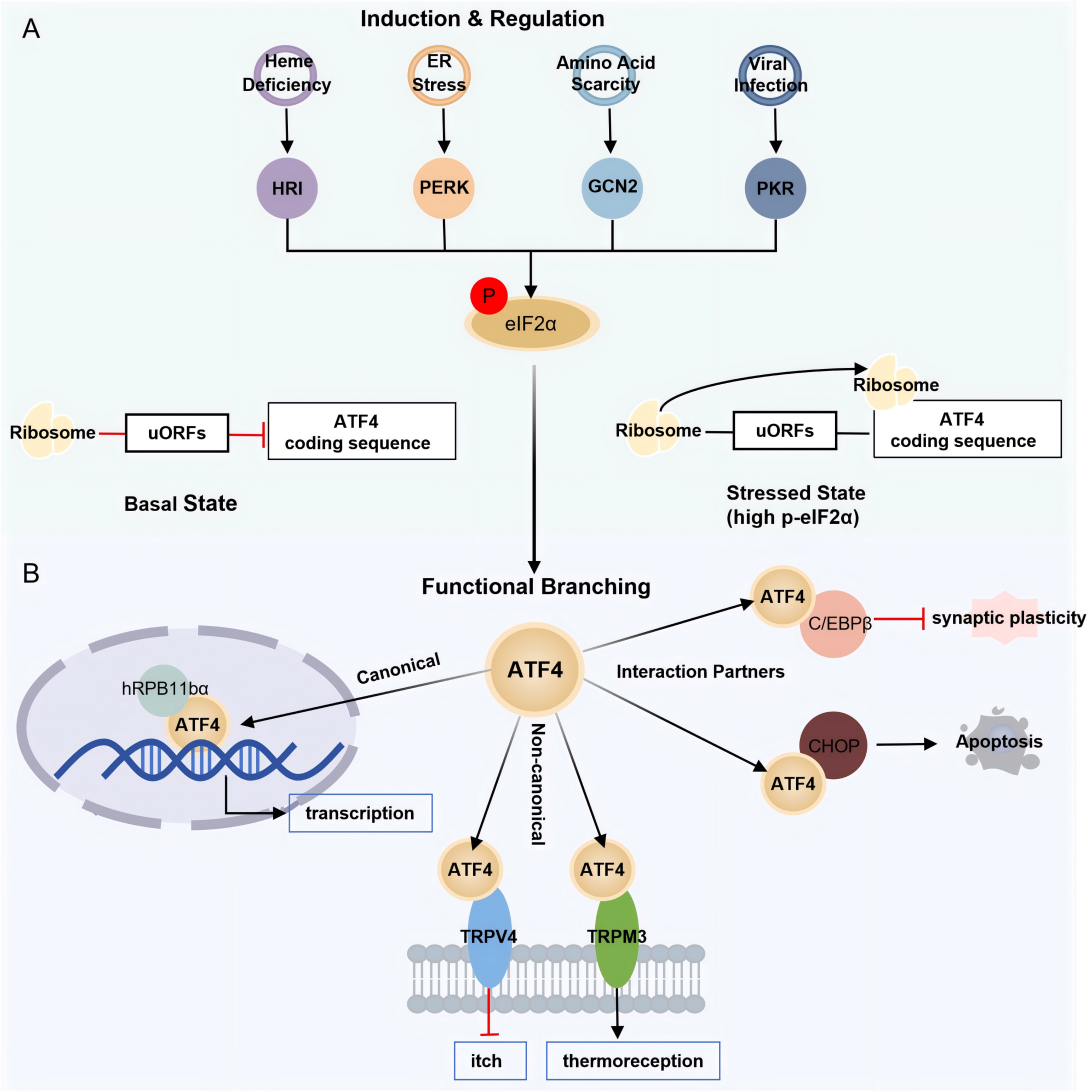
The multi-layered regulatory landscape and functional branching of ATF4. **(A)** Induction and translational gating. Diverse homeostatic disruptions activate four respective eIF2α kinases (HRI, PERK, GCN2, and PKR), converging on eIF2α phosphorylation (p-eIF2α). Under basal conditions (left), inhibitory upstream open reading frames (uORFs) prevent the translation of the ATF4 coding sequence. During stress (right), p-eIF2α enables ribosomes to bypass these elements, selectively initiating ATF4 synthesis. **(B)** Functional branching and molecular interactions. Canonical transcription: ATF4 translocates to the nucleus, where hRPB11bα anchors it to the RNA Polymerase II machinery to refine transcriptional specificity. Interactome fate decisions: The biological output depends on dimerization stoichiometry. CHOP-dimerization promotes apoptosis, while competitive antagonism with CREB and cooperation with C/EBPβ act as homeostatic gates for synaptic reset and resilience. Non-canonical execution: ATF4 exerts immediate, transcription-independent effects by silencing TRPV4 or chaperoning TRPM3 on the plasma membrane, acutely recalibrating neuronal excitability. Black arrows: promotion; red T-bars: inhibition.

Activating transcription factor 4 serves as a ubiquitous stress sensor, with expression induced by insults ranging from hypoxia and nutrient deprivation to proteotoxic stress (Vattem and Wek, 2004). Central to this response is the PERK-eIF2α signaling axis. Upon the accumulation of misfolded proteins, PERK autophosphorylation triggers eIF2α phosphorylation, which selectively shifts the translational machinery toward ATF4 mRNA via upstream open reading frames (uORFs) while dampening global protein synthesis (Harding et al., 2000; Szegezdi et al., 2006). The pathological relevance of this pathway is evident in Parkinson’s disease models, where chronic recruitment of the PERK-eIF2α-ATF4 axis by neurotoxins, such as 1-methyl-4-phenylpyridinium (MPP^+^) and 6-hydroxydopamine (6-OHDA), drives an apoptotic program mediated by C/EBP homologous protein (CHOP), tribbles homolog 3 (TRIB3), and P53 upregulated modulator of apoptosis (Puma) (Demmings et al., 2021; Go et al., 2017; Wang et al., 2020). While PERK is the primary ER transducer, other ISR kinases—general control non-derepressible 2 (GCN2), protein kinase R (PKR), and heme-regulated inhibitor (HRI)—confer ATF4 responsiveness to amino acid scarcity, viral infection, and heme deficiency, respectively (Kalinin et al., 2025; Masson, 2019; Nakagawa and Ohta, 2019; Yuan et al., 2020; Figure 1A). Recent evidence further integrates this axis into metabolic networks. For example, following intracerebral hemorrhage, the PERK-ATF4 branch couples with mitochondrial one-carbon metabolism via methylenetetrahydrofolate dehydrogenase 2 (MTHFD2). This feedback loop facilitates mitochondrial redox buffering and purine biosynthesis, thereby bolstering neuronal antioxidant capacity against oxidative injury (Liu et al., 2024).

### 2.2 The multilayered regulatory landscape and interaction partners of ATF4

The functional versatility of ATF4 is governed by a regulatory architecture spanning translational, post-translational, and protein-protein interaction domains. At the translational level, ATF4 synthesis is controlled by a re-initiation mechanism involving uORFs. Under stress, eIF2α phosphorylation facilitates ribosomal bypassing of inhibitory uORF2, triggering the preferential translation of the ATF4 coding sequence (Vattem and Wek, 2004; Wek et al., 2023; Figure 1B).

Beyond its synthesis, the transactivation potency of ATF4 is fundamentally modulated by its transcriptional interactors, which dictate the downstream response and the survival-apoptosis balance. ATF4 can form homodimers or heterodimers with varied basic leucine zipper (b-Zip) partners; for instance, dimerization with CHOP promotes maladaptive apoptosis (Demmings et al., 2021), whereas its cooperative dimerization with CCAAT/enhancer-binding protein beta (C/EBPβ) and competitive antagonism with cAMP-response element-binding protein (CREB) function as critical homeostatic gates for synaptic plasticity and neuronal resilience (Costa-Mattioli et al., 2007; Sun et al., 2013). Furthermore, recruitment by subunits such as hRPB11bα (a component of the RNA polymerase II core) anchors ATF4 to the RNA polymerase II machinery, refining its transcriptional specificity (Proshkin et al., 2019). Intriguingly, ATF4 also exerts non-transcriptional control over neuronal excitability; it directly associates with and silences transient receptor potential vanilloid 4 (TRPV4) activity or chaperones transient receptor potential melastatin 3 (TRPM3) to the plasma membrane, thereby gating sensory signaling independent of nuclear translocation (Xie et al., 2024, 2021). The stability of these complexes is tightly coupled to the ubiquitin-proteasome system (UPS), which sets the threshold for gene expression essential for long-term synaptic plasticity (Smith et al., 2020; Wu et al., 2010; Figure 1B).

### 2.3 Cell-type-specific ATF4 programs: a framework for neuronal resilience

Activating transcription factor 4 acts as a “dynamic signal processor” whose output is tailored to specific neuronal identities. This cell-type specificity is exemplified by the divergent roles of ATF4 in excitatory versus inhibitory lineages. In excitatory neurons, ATF4 imposes a bioenergetic limit on synaptic potency (Costa-Mattioli et al., 2007; Mahmood et al., 2024), whereas its role in cognitive gating within inhibitory circuits appears distinct. These specific programs balance metabolic robustness against functional demand. For example, the heightened vulnerability of midbrain dopaminergic neurons to ISR-driven apoptosis reflects a lower threshold for ATF4-mediated death signaling, partially due to their inherent oxidative load (Demmings et al., 2021; Li P. et al., 2023; Sun et al., 2013). Conversely, hippocampal neurons utilize ATF4 to fine-tune intrinsic excitability via gamma-aminobutyric acid (GABA)ergic signaling, providing a critical buffer against excitotoxicity and seizure-induced damage (Corona et al., 2018; Yu et al., 2023). Such diversity suggests that the “rheostat setting” of ATF4 is cell-type-determined, necessitating a transition from broad ISR inhibition toward precision, circuit-based therapeutic interventions that respect these innate biological thresholds.

## 3 Determining neuronal fate: the dualistic nature of ATF4

Activating transcription factor 4 integrates signals from the ER, mitochondria, and metabolic pools to dictate divergent neuronal fates. This bimodal output functions as a “threshold-gated” rheostat: while transient induction engages reparative networks, chronic stress shifts the interactome stoichiometry toward terminal failure. Thus, the survival-death equilibrium hinges on the duration of stress and the specific recruitment of transcriptional partners.

### 3.1 Pro-survival signaling: homeostatic buffering and repair

Under adaptive stress, ATF4 mobilizes a suite of evolutionary conserved survival pathways designed to restore cellular equilibrium. This reparative phase is characterized by a concerted expansion of proteostatic capacity, where ATF4 transactivates chaperones like binding immunoglobulin protein (BiP/GRP78) to enhance luminal folding and engages protective autophagy to mitigate the accumulation of proteotoxic aggregates (Chu et al., 2023; Costa-Mattioli and Walter, 2020; Harding et al., 2003, 2002; Siu et al., 2002). Beyond the endoplasmic reticulum, ATF4 orchestrates a robust antioxidant and metabolic defense by upregulating solute carrier family 7 member 11 (SLC7A11) and the heme oxygenase-1 (HO-1) axis. The upregulation of these targets sustains intracellular glutathione levels and mitigates ferroptotic cell death, thereby sustaining glutathione levels and bioenergetic stability (Almeida et al., 2022; Dey et al., 2015; Inoue et al., 2018; Iqbal et al., 2024). This adaptive network further extends to mitochondrial surveillance through the induction of the mitochondrial unfolded protein response (mtUPR), which recruits proteases such as caseinolytic mitochondrial matrix peptidase proteolytic subunit (ClpP) and chaperones like heat shock protein 60 (HSP60) to preserve organellar integrity under acute insults (Yu et al., 2023). Together, these coordinated responses define an “adaptive window” where ATF4 actively recalibrates neuronal metabolism to avert terminal cellular collapse.

### 3.2 Pro-apoptotic mechanisms: the transition to terminal failure

When cellular stress exceeds homeostatic buffers, the ATF4 interactome undergoes a pathological pivot. The hallmark of this transition is the sustained induction of CHOP, which displaces pro-survival co-factors to form ATF4-CHOP heterodimers (Iurlaro and Muñoz-Pinedo, 2016). This complex serves as a terminal node that executes a lethal repertoire of B-cell lymphoma 2 (BCL-2) family members—including BCL-2-like protein 11 (Bim), death receptor 5 (DR5), and PUMA—to trigger mitochondrial outer membrane permeabilization and caspase activation (Demmings et al., 2021; Puthalakath et al., 2007). Maladaptive ATF4 signaling further amplifies neurotoxicity through context-specific pathways, such as tribbles homolog 3 (TRIB3)-mediated Parkin depletion, jumonji domain-containing protein 3 (JMJD3)-dependent upregulation of transcription factor jun-B (JUNB) and protein C-ets-1 (ETS1), and synergistic ER stress exacerbation with TAR DNA-binding protein 43 (TDP-43; Luan et al., 2023; Wang et al., 2024; Wu et al., 2022). Beyond classical apoptosis, chronic hyperactivation facilitates ferroptotic cascades via the epigenetic suppression of glutathione peroxidase 4 (GPX4; Lu et al., 2025; Yin et al., 2025). This “locked-in” feed-forward loop marks the irreversible exhaustion of neuronal resilience and the transition to pathogenic decay.

## 4 From developmental scaffolding to synaptic homeostasis

**FIGURE 2:**
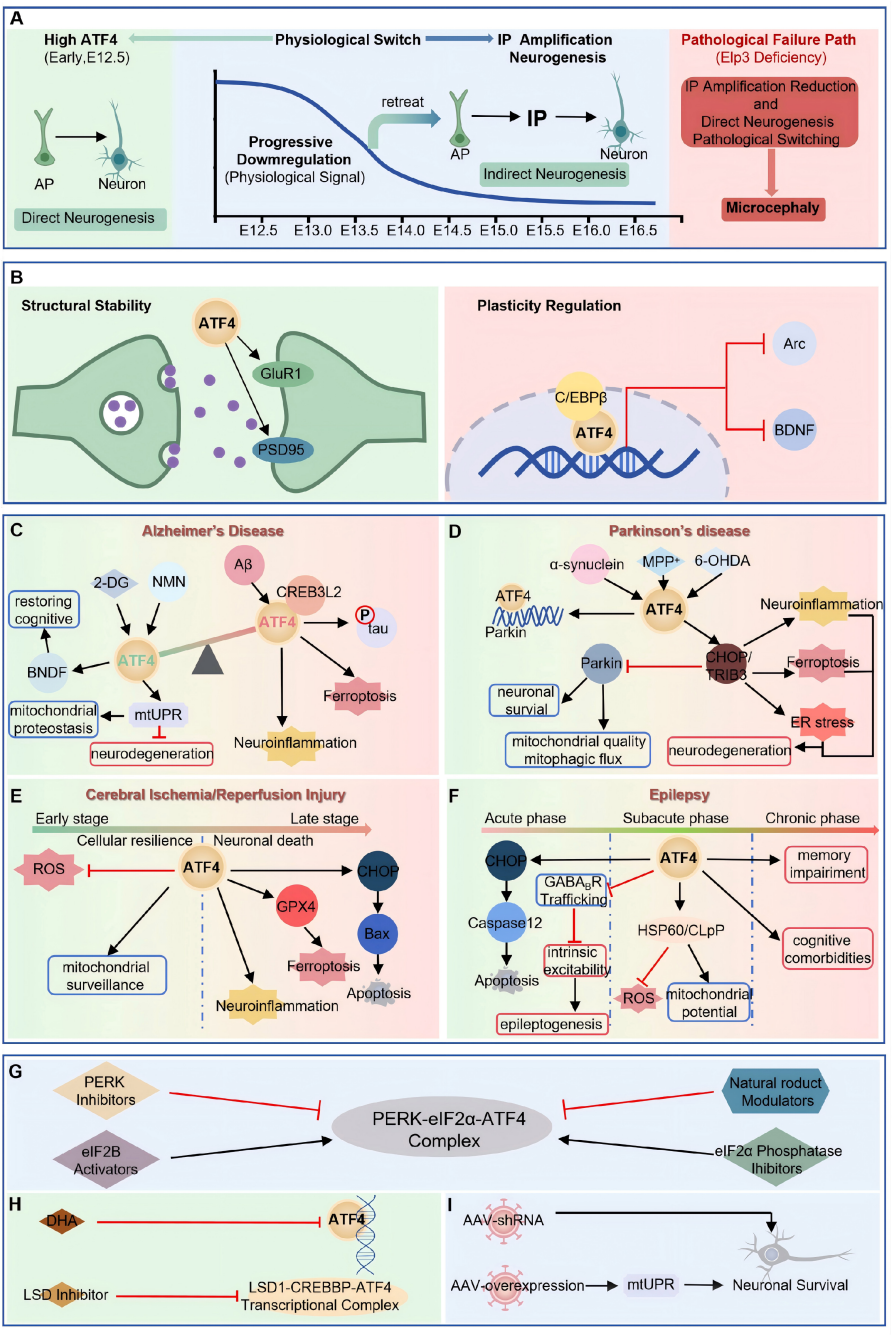
Activating transcription factor 4 (ATF4) as a spatiotemporal rheostat in brain health and disease. **(A)** Neocortical morphogenesis. During corticogenesis (E12.5–E16.5), a programmed “ATF4 retreat” acts as a developmental switch. High early-stage ATF4 drives direct neurogenesis, while its downregulation is required for IP expansion and cortical scaling. Pathological persistence leads to IP depletion and microcephaly. **(B)** Synaptic homeostasis. In mature neurons, ATF4 regulates PSD95 and GluR1 for structural stability. As a molecular brake, it cooperates with C/EBPβ to repress plasticity-related genes (Arc, BDNF), setting the threshold for synaptic scaling and LTP. **(C–F)** Bimodal pathogenesis in neurological disorders. Neuronal fate is determined by the duration and intensity of ATF4 activation. **(C)** Alzheimer’s disease: Pathological Aβ42 drives the ATF4-CREB3L2 axis, causing Tau phosphorylation and ferroptosis. Adaptive metabolic priming (NMN/2-DG) recruits ATF4 for mitochondrial protection. **(D)** Parkinson’s disease: A temporal shift from early Parkin-mediated mitophagy to late-stage CHOP/TRIB3-mediated Parkin suppression and cell death. **(E)** Cerebral ischemia/reperfusion: A pivot from early antioxidant resilience to late-stage GPX4-dependent ferroptosis and apoptosis. **(F)** Epilepsy: Acute excitotoxicity triggers caspase-12 activation and GABABR internalization; chronic elevation leads to memory impairment. **(G–I)** Integrated therapeutic strategies. **(G)** ISR calibration: PERK inhibitors quench maladaptive signaling. eIF2B activators act as “ISR desensitizers” by stabilizing the eIF2B decamer to maintain protein synthesis despite p-eIF2α accumulation. Conversely, eIF2α phosphatase inhibitors sustain p-eIF2α levels to prolong adaptive resilience. Natural product modulators provide further pleiotropic fine-tuning of the ISR-ATF4 complex. **(H)** Transcriptional interactome disruption: Targeted decoupling of ATF4 from DNA-binding sites (via DHA) or pharmacological disassembly of specific co-regulator scaffolds (e.g., the LSD1-CREBBP-ATF4 complex) enables a “molecular scalpel” approach to silencing pathogenic outputs. **(I)** Precision gene therapy: AAV-shRNA enables targeted ATF4 silencing to rescue dopaminergic loss in PD, whereas AAV-overexpression recruits mtUPR to fortify mitochondrial robustness in epilepsy. Black arrows: promotion; red T-bars: inhibition. Detailed agent data are provided in Table 1.

While the role of ATF4 is often synonymous with stress, it functions as a foundational governor of neuronal ontogeny and circuit refinement. In the nascent neocortex, the eIF2α-ATF4 axis acts as a “specification switch” for neuronal lineage commitment. As demonstrated by Laguesse et al. (2015), a programmed reduction of ATF4 in apical progenitors between embryonic days 12.5 (E12.5) and 16.5 (E16.5) is essential for the transition from direct to indirect neurogenesis. Disrupting this temporal decline—as seen in elongator complex protein 3 (Elp3)-deficient models—leads to premature progenitor exhaustion and microcephaly, establishing the rhythmic “retreat” of ATF4 signaling as a prerequisite for proper brain assembly (Laguesse et al., 2015; Figure 2A).

Beyond development, ATF4 serves as a pivotal arbiter of neural circuit refinement and cognitive output. Under physiological conditions, it traditionally functions as a “memory suppressor”; its targeted ablation in forebrain excitatory neurons bolsters long-term memory and lowers the threshold for long-term potentiation (LTP) induction. This effect is attributed to the release of transcriptional brakes on oxidative phosphorylation and adenosine triphosphate (ATP) bioavailability, thereby enhancing the metabolic headroom available for synaptic strengthening (Chen et al., 2003; Costa-Mattioli et al., 2005). However, this inhibitory role is counterbalanced by its requirement for structural integrity. ATF4 knockdown precipitates the loss of dendritic spines and postsynaptic scaffolds, including glutamate ionotropic receptor AMPA type subunit 1 (GluR1) and postsynaptic density protein 95 (PSD95; Liu et al., 2014), suggesting that ATF4 maintains the “synaptic reset” necessary for homeostatic scaling (Amar et al., 2021; Pasini et al., 2015). Mechanistically, ATF4 coordinates with C/EBPβ to repress plasticity-related genes such as activity-regulated cytoskeleton-associated protein (Arc) and brain-derived neurotrophic factor (BDNF; Bartsch et al., 1995; Chen et al., 2003; Pavlopoulos et al., 2013), while simultaneously engaging epigenetic modulators like JMJD3 to prime pro-regenerative programs (Weng et al., 2018; Figure 2B). The pathological subversion of this balance is a hallmark of neuropsychiatric dysfunction. For instance, DISC1-mediated regulation of ATF4 nuclear stoichiometry is critical for synaptic maintenance; its disruption leads to aberrant ATF4 accumulation and subsequent synaptic collapse, a phenotype partially reversible by ATF4 titration (Malavasi et al., 2012; Soda et al., 2013; Wang et al., 2021).

## 5 The pathological nexus: ATF4 orchestrates disease evolution

While transient ATF4 signaling preserves neuronal integrity, its chronic subversion acts as a pathological rheostat that catalyzes the transition from homeostatic resilience to irreversible circuit decay. The following sections detail how diverse pathological triggers hijack this axis to drive the progression of neurodegenerative and functional disorders.

### 5.1 Alzheimer’s disease (AD)

In AD, ATF4 operates as a molecular arbiter whose output critically sways between resilience and decay. Pathologically, β-amyloid-42 (Aβ_42_) promotes the heterodimerization of ATF4 with CREB3L2 by impairing proteasomal flux; this complex acts as a transcriptional hub governing nearly half of AD-specific differentially expressed genes, directly catalyzing tau hyperphosphorylation (Herline-Killian et al., 2025; Shao et al., 2023; Wei et al., 2015). Furthermore, the PERK-ATF4 axis facilitates a maladaptive transition toward ferroptosis and neuroinflammation, correlated with eIF2α-dependent ATF4 activation in AD patients (Currais et al., 2025; Shao et al., 2023; Zhang et al., 2024). Paradoxically, ATF4 also harbors a neuroprotective potential. Moderate ISR mobilization recruits ATF4 to transactivate pro-plasticity factors such as BDNF, while nicotinamide adenine dinucleotide (NAD^+^) precursors utilize the ATF4-dependent mtUPR to fortify mitochondrial proteostasis, thereby enhancing ATP production and neuronal survival (Kumar et al., 2023; Xiong et al., 2024). Thus, the net impact of ATF4 in AD is dictated by the kinetics and microenvironmental context of its induction (Figure 2C).

### 5.2 Parkinson’s disease (PD)

In PD, ATF4 operates as a decisive molecular switch tuned by the chronicity of cellular stress. The neurotoxic facet of ATF4 is tied to the sustained activation of the ATF4/CHOP-TRIB3-Parkin axis. Under persistent proteostatic insult—triggered by α-synuclein or neurotoxins like MPP^+^ and 6-OHDA—the ATF4/CHOP heterodimer drives the upregulation of TRIB3. This recruitment directly depletes the E3 ubiquitin ligase Parkin, thereby impairing mitochondrial clearance and precipitating dopaminergic neuronal attrition (Aimé et al., 2020, 2015; Demmings et al., 2021). Conversely, ATF4 harbors intrinsic neuroprotective potential under adaptive stress regimes, where it functions as a direct transcriptional activator of Parkin, binding to its promoter to bolster mitophagy and mitochondrial quality control (Bouman et al., 2011; Gully et al., 2016; Ham et al., 2023; Wu et al., 2013). Ultimately, the fate of neurons hinges on whether the ATF4 network favors the pro-apoptotic CHOP/TRIB3 cascade or the adaptive Parkin-mediated repair program (Figure 2D).

### 5.3 Cerebral ischemia/reperfusion injury

Activating transcription factor 4 in cerebral ischemia is characterized by a tipping point between early-stage resilience and late-stage neurotoxicity. Initially, moderate ATF4 signaling orchestrates protective programs—including mitophagic flux and antioxidant defense—to buffer acute bioenergetic collapse (He et al., 2019; Li Y. et al., 2023). However, the persistent “overdrive” of the axis becomes deleterious, marked by the assembly of ATF4-CHOP complexes that drive Bax-mediated apoptosis and the epigenetic suppression of the ferroptosis-protector GPX4 (Bai et al., 2022; Hadley et al., 2018; Lu et al., 2025; Yin et al., 2025). Successful pharmacological targeting relies on an exquisite understanding of these activation kinetics to “remodel” rather than simply “block” the ATF4 response (Figure 2E).

### 5.4 Epilepsy

In the epileptic brain, ATF4 functions as a dynamic node that oscillates across disease stages. During seizure onset, the PERK-eIF2α-ATF4 response is predominantly maladaptive; ATF4 orchestrates a transcriptional program favoring CHOP and caspase12-mediated apoptosis (Cui et al., 2024; Doğanyiğit et al., 2023; Kim et al., 2014). Crucially, ATF4 extends its reach to the synapse, erodes inhibitory tone by destabilizing cell division control protein 42 homolog (Cdc42), which impairs the membrane trafficking of gamma-aminobutyric acid type B receptor (GABA_*B*_R), thereby reducing inhibitory synaptic strength and facilitating epileptogenesis (Corona et al., 2018). Paradoxically, in the subacute phase, ATF4 pivots toward mitochondrial surveillance by upregulating HSP60 and ClpP to restore mitochondrial potential (Yu et al., 2023). This spatiotemporal duality mandates that interventions focus on the “kinetic recalibration” of its signaling flux (Figure 2F).

## 6 Therapeutic targeting of the ATF4 axis: from inhibition to precision modulation

The functional dichotomy of ATF4—governing both neuroprotective adaptation and terminal decay—presents a “therapeutic paradox” that complicates pharmacological intervention. While ATF4 remains a high-value target at the epicenter of the ISR, clinical success requires a transition from broad-spectrum inhibition toward biomarker-guided, precision modulation. The following strategies evaluate current efforts to harness ATF4’s protective potential while circumventing its maladaptive “toxic face.”

### 6.1 Recalibrating upstream ISR signaling cascades

The most pharmacologically advanced strategy targets the upstream kinases and phosphatases that gate eIF2aα phosphorylation. To mitigate the pathological “overdrive” of the ISR, selective PERK inhibitors—such as GSK2606414 and AMG-44—effectively quench maladaptive ATF4 transactivation, curbing apoptosis in models of Alzheimer’s, Parkinson’s and cerebral ischemia (Dhir et al., 2023; Mercado et al., 2018; Radford et al., 2015). Conversely, in contexts where ATF4-driven adaptation is beneficial, phosphatase inhibitors like salubrinal bolster resilience by sustaining p-eIF2α levels (Wu et al., 2014; Figure 2G).

A paradigm-shifting addition to this toolkit is the development of eIF2B activators (e.g., integrated stress response inhibitor (ISRIB) and its clinical-grade derivative DNL343), which function as “ISR desensitizers.” These molecules stabilize the eIF2B decamer to maintain protein synthesis even in the presence of p-eIF2α. While DNL343 demonstrated robust preclinical neuroprotection and reached phase 1 safety benchmarks (Craig et al., 2024; Flores et al., 2025; Yulyaningsih et al., 2024), both DNL343 (Denali) and ABBV-CLS-7262 (Calico/AbbVie) faced critical setbacks in January 2025 clinical trials for amyotrophic lateral sclerosis (ALS), failing to meet primary efficacy endpoints (Jeong et al., 2025). These failures underscore a fundamental “therapeutic paradox”: wholesale ISR suppression may inadvertently dismantle essential homeostatic shields. As metabolic states dictate ISR activation mechanisms, absolute blockade can impair vital adaptive responses, such as the OMA1-DELE1-HRI axis-comprising overlapping with m-AAA protease 1 (OMA1), DAP5-associated protein 1 (DELE1), and heme-regulated inhibitor (HRI)-which is essential for mitochondrial surveillance (Jeong et al., 2025). Furthermore, while ISR inhibition is therapeutic in neurodegeneration, it may also strip glioblastoma cells of their stress-adaptive shields, offering a potential strategy to circumvent chemoresistance (Lan et al., 2024). Collectively, these divergent outcomes necessitate a transition from generic inhibition toward “kinetic recalibration”—ensuring that interventions specifically target the window of pathological commitment without compromising physiological resilience. The current landscape of ATF4-targeted interventions, categorized by their regulatory node and translational hurdles, is summarized in Table 1. These diverse approaches reflect the ongoing effort to shift from global pathway blockade toward the “molecular scalpel” paradigm.

**TABLE 1:** Representative modulators and therapeutic strategies targeting the ISR-ATF4 axis.

Class/target	Example compounds	Mechanism of action	Primary effect on ATF4	Therapeutic context/note
PERK inhibitors	GSK2606414, AMG-44	Inhibit PERK kinase activity, reducing eIF2α phosphorylation.	Suppress induction	Neurodegeneration (AD, PD), cerebral ischemia. May impair adaptive ISR.
eIF2α phosphatase inhibitors	Salubrinal	Inhibit dephosphorylation of p-eIF2α, prolonging its activity.	Enhance/sustain induction	Proteotoxic stress models, to boost adaptive responses.
eIF2B activators	ISRIB, DNL343, ABBV-CLS-7262	Stabilize eIF2B decamer, restoring translation initiation despite p-eIF2α.	Bypass/desensitize	Designed to counteract chronic ISR; faced efficacy challenges in ALS trials.
Transcriptional complex disruptors	(e.g., DHA, LSD1 inhibitors)	Interfere with ATF4-DNA binding or co-factor assembly.	Alter transcriptional specificity	Emerging strategy for precision targeting (e.g., in oncology).
Natural product modulators	Curcumin, Resveratrol, Apigenin	Multi-target, often via upstream signaling or direct interaction.	Context-dependent inhibition	Neuroprotection, adjunctive therapy with antioxidant benefits.
Gene therapies	AAV-shRNA, Lentivirus	Viral-mediated silencing (shRNA) or overexpression of ATF4.	Level recalibration	Offers exquisite spatiotemporal control; proven in PD (silencing) and Epilepsy (induction)

### 6.2 Disrupting the ATF4 transcriptional interactome

Directly perturbing the protein-protein or protein-DNA interfaces of ATF4 offers a pathway to highly specific therapeutic outcomes, albeit with the challenges inherent in targeting intrinsically disordered transcription factors. Docosahexaenoic acid (DHA) exemplifies this potential; by decoupling ATF4 from target promoters such as SLC7A11, DHA triggers ferroptotic cascades in oncogenic contexts, establishing a mechanistic precedent for the development of small-molecule ATF4-DNA binding inhibitors (Tang N. et al., 2024). A more nuanced strategy involves the pharmacological disassembly of multi-protein scaffolds. In glioblastoma models, targeting the histone demethylase lysine-specific demethylase 1 (LSD1) effectively shatters the LSD1-CREBBP-ATF4 transcriptional complex, selectively silencing the stress-adaptive programs essential for tumor survival (Faletti et al., 2021; Figure 2H). These interventions highlight a transition from global ISR inhibition toward the “molecular scalpel” approach—targeting specific ATF4-cofactor assemblies to achieve exquisite functional control while minimizing collateral disruption of homeostatic signaling.

### 6.3 Natural products as multimodal regulators of ATF4 signaling

Natural products offer a unique pharmacopeia of multimodal regulators capable of fine-tuning the ATF4 rheostat without the “all-or-nothing” toxicity of synthetic inhibitors. Pleiotropic compounds—including curcumin, resveratrol, and apigenin—provide a multi-pronged defense by modulating ATF4 expression and activity in a context-dependent manner (Gu et al., 2021; Ji et al., 2025; Luo et al., 2023). A notable example is adiponectin peptides, which prevent apoptosis by uncoupling the mothers against decapentaplegic homolog 3 (Smad3)-ATF4-CHOP axis under metabolic stress (Wu et al., 2020). Characterized by favorable safety profiles and multi-target engagement, these natural scaffolds serve as sophisticated lead structures for drug discovery, offering a “gentle” modulation strategy essential for the long-term management of chronic neurodegeneration, where maintaining baseline homeostatic signaling is as critical as suppressing pathological peaks (Figure 2G).

### 6.4 Precise spatiotemporal control via gene therapy modalities

The dual-role biology of ATF4 mandates a transition toward gene-based interventions that offer exquisite spatiotemporal specificity. The success of adeno-associated virus (AAV)-delivered short hairpin RNA (shRNA)-mediated ATF4 silencing in mitigating dopaminergic loss (Demmings et al., 2021) and lentiviral overexpression to bolster mitochondrial robustness in epilepsy (Yu et al., 2023) underscores the versatility of genetic manipulation (Figure 2I). Unlike systemic small molecules, gene therapy facilitates the “functional partitioning” of the ATF4 response—enabling site-specific suppression in degenerating neurons while preserving adaptive signaling in healthy circuits. As viral vector technology incorporates cell-type-specific enhancers and refined delivery routes, gene therapy represents the most durable modality for translating ATF4’s complex dynamics into circuit-specific clinical interventions.

## 7 Discussion: ATF4 as a spatiotemporal rheostat and pathogenic switch

Activating transcription factor 4 emerges not as a binary switch for neuronal survival, but as the central transcriptional processor of the ISR, governing a bimodal output that calibrates neuronal fate. Its function is best conceptualized as a spatiotemporal rheostat, whose setting is dynamically adjusted by developmental timing, subcellular context, and the kinetics of proteostatic flux. This regulatory sophistication is evident from the nascent neocortex—where ATF4 acts as a specification switch for neurogenesis—to the adult synapse, where it maintains homeostatic scaling. These foundational roles establish ATF4 as a fundamental governor of brain architecture and cognitive plasticity, far beyond a mere respondent to injury.

The pathogenic cascade across neurodegenerative, ischemic, and epileptogenic disorders represents a maladaptive hijacking of this precise logic. The transition from resilience to decay is marked by a “locked-in” hyperactivation of the ATF4-CHOP axis, fueling ferroptosis and synaptic collapse. However, this destructive phase exists in chronic tension with ATF4’s indispensable role in mobilizing adaptive shields, such as mitochondrial surveillance (via the OMA1-DELE1-HRI axis) and the compensatory network stabilization observed in epilepsy models. This intrinsic duality creates a “therapeutic paradox”: wholesale suppression of ATF4 may curb apoptosis but risks dismantling the very homeostatic buffers—particularly those preserving organellar robustness—required for neuronal survival.

The recent clinical failures of ISR modulators (e.g., DNL343 and ABBV-CLS-7262) in 2025 ALS trials provide a stark validation of this paradox. These setbacks underscore that broad-spectrum blockade is an inadequate strategy that overlooks the “threshold logic” of the ISR. Consequently, the future of targeting this axis necessitates a paradigm shift from global inhibition toward “kinetic recalibration.” The objective is to develop precision interventions—leveraging molecular scalpels to disrupt specific interactomes or gene therapies for circuit-specific control—that can restore homeostatic equilibrium without ablating vital physiological functions.

To realize this vision, future research must resolve the cell-type-specific “tipping points” of the ATF4 rheostat. Elucidating how local metabolic states and epigenetic landscapes dictate the bimodal output of ATF4 will be essential. By advancing from mere stress suppression to mastering the information flux of the ISR network, next-generation therapies can aim to restore the long-term computational homeostasis of the nervous system, offering durable interventions for a spectrum of neurological diseases.
